# ARC: An Open Web-Platform for Request/Supply Matching for a Prioritized and Controlled COVID-19 Response

**DOI:** 10.3389/fpubh.2021.607677

**Published:** 2021-02-16

**Authors:** Jean-Denis Courcol, Cédric F. Invernizzi, Zachary C. Landry, Mikhaél Minisini, Dieter A. Baumgartner, Sebastian Bonhoeffer, Barbara Chabriw, Estelle E. Clerc, Michael Daniels, Pavlo Getta, Matthieu Girod, Kinga Kazala, Henry Markram, Axel Pasqualini, Clara Martínez-Pérez, François J. Peaudecerf, Margit S. Peaudecerf, Ulrike Pfreundt, Benjamin R. K. Roller, Jonasz Słomka, Marie Vasse, Jeanette D. Wheeler, César M. J. A. Metzger, Roman Stocker, Felix Schürmann

**Affiliations:** ^1^Blue Brain Project, Ecole polytechnique fédérale de Lausanne (EPFL), Geneva, Switzerland; ^2^Spiez Laboratory, Federal Office for Civil Protection FOCP, Spiez, Switzerland; ^3^Environmental Microfluidics Group, Institute of Environmental Engineering, Department of Civil, Environmental and Geomatic Engineering, ETH Zürich, Zurich, Switzerland; ^4^Apptitude SA, Lausanne, Switzerland; ^5^Institute for Integrative Biology, Department of Environmental Systems Science, ETH Zürich, Zurich, Switzerland; ^6^Institute of Biogeochemistry and Pollutant Dynamics, Department of Environmental Systems Science, ETH Zürich, Zurich, Switzerland; ^7^Department of Environmental Microbiology, Eawag, Dübendorf, Switzerland; ^8^Section Software Services, Department of IT Services, ETH Zürich, Zurich, Switzerland; ^9^Institute for Biochemistry, Department of Biology, ETH Zürich, Zurich, Switzerland

**Keywords:** COVID-19, crisis response, diagnostic supplies, customizable match-making web platform, prioritization, open source, easy deployment

## Abstract

In 2020 the world was hit by the COVID-19 pandemic putting entire governments and civil societies in crisis mode. Around the globe unprecedented shortages of equipment and qualified personnel were reported in hospitals and diagnostic laboratories. When a crisis is global, supply chains are strained worldwide and external help may not be readily available. In Switzerland, as part of the efforts of the Swiss National COVID-19 Science Task Force, we developed a tailor-made web-based tool where needs and offers for critical laboratory equipment and expertise can be brought together, coordinated, prioritized, and validated. This Academic Resources for COVID-19 (ARC) Platform presents the specialized needs of diagnostic laboratories to academic research groups at universities, allowing the sourcing of said needs from unconventional supply channels, while keeping the entities tasked with coordination of the crisis response in control of each part of the process. An instance of the ARC Platform is operated in Switzerland (arc.epfl.ch) catering to the diagnostic efforts in Switzerland and sourcing from the Swiss academic sector. The underlying technology has been released as open source so that others can adopt the customizable web-platform for need/supply match-making in their own relief efforts, during the COVID-19 pandemic or any future disaster.

## 1. Introduction

The COVID-19 pandemic first reached Switzerland on February 25th 2020, when the first Swiss case was detected. The person was placed in isolation and officially confirmed as infected (1). Three days later the first pandemic management measure (prohibition of gatherings of >1,000 persons) was implemented by federal decree (2). Within three weeks, the case load rose to >1,000 new cases per day and a cumulative total of >5,000 confirmed infected patients (3). The pandemic became a societal challenge on many levels.

During this first wave of the COVID-19 pandemic in Switzerland, the Swiss federal government set up procedures for the prioritization, emergency procurement and distribution of certain goods and equipment urgently needed by the healthcare system. At the same time, it recognized the need for early detection of shortcomings in the supply chain and shortages of personnel in the medical microbiology laboratories responsible for diagnostic testing. Subsequently, the Swiss Federal Institute for Nuclear, Biological and Chemical (NBC) Protection (Spiez Laboratory) was mandated by the Swiss Federal Council (the highest central executive government authority in Switzerland) to establish a system to monitor the situation in the testing laboratories and to identify bottlenecks and critical issues in their supply chain (4).

Concomitantly, as the number of cases rose, the first bottlenecks became apparent. During a pandemic, healthcare and laboratory supply chains are highly dependent on the situation in other countries. Around the world there were reports of unprecedented shortages in personal protective equipment, swabs for hospitals, molecular biology equipment, consumables for testing laboratories, as well as hospital and laboratory staff (5–7).

Since it was proving difficult to address the emerging bottlenecks through healthcare and laboratory supply companies, the organizations responsible for the Swiss crisis response turned to complementary solutions. One was the creation of new collaborative networks. These included a new network linking federal government laboratories [Spiez Laboratory, Agroscope, the Institute of Virology and Immunology (IVI), the Swiss Federal Institute of Aquatic Science and Technology (Eawag), the Swiss Federal Institute for Forest, Snow and Landscape Research (WSL), the Federal Institute of Metrology (METAS), Swiss Agency for Therapeutic Products (Swissmedic), the Federal Food Safety and Veterinary Office (FSVO), the Paul Scherrer Institute (PSI), armasuisse Science and Technology S+T] and diagnostic laboratories. Members of the network were able to exchange or loan analytic equipment (such as lightcyclers or extraction robots), consumables, protective equipment, and sometimes qualified personnel.

In parallel, the Swiss academic sphere, who at that point was hampered in its operations due to the partial lockdown measures imposed as of March 16th by the government to control the pandemic, started working on diverse initiatives to contribute to the national pandemic management and relief efforts. In many countries scientific facilities and staff have been repurposed to fight the pandemic. Research laboratories were turned into SARS-CoV-2 testing laboratories, scientists were sent to support diagnostic laboratories in hospitals, and (parts of) hospitals were transformed into isolation wards. Scientists from the Francis Crick Institute have published a roadmap for other institutes based on their efforts in (8).

In Switzerland, the crisis also led to the creation of the Swiss National COVID-19 Science Task Force (NCS-TF, see www.ncs-tf.ch) to advise the Federal Council during the pandemic. The task force originated from the ETH Domain (i.e., the network of Swiss federal universities and research institutions), and subsequently expanded to include academics from nearly all Swiss universities as well as other scientists. The mandate of the task force is to provide the Swiss Federal Office of Public Health (FOPH) and the Swiss Federal Department of Home Affairs (FDHA) with its scientific knowledge to inform and support political authorities and decision-makers. Additionally, the task force works toward identifying fields and opportunities for research and innovation where scientific know-how can contribute to COVID-19-related products or services. One example is the helpful ETH platform (helpful.ethz.ch), which proposes the use of academic research groups' capabilities to produce certain goods, for instance by 3D-printing personal protective equipment.

**Figure 1:**
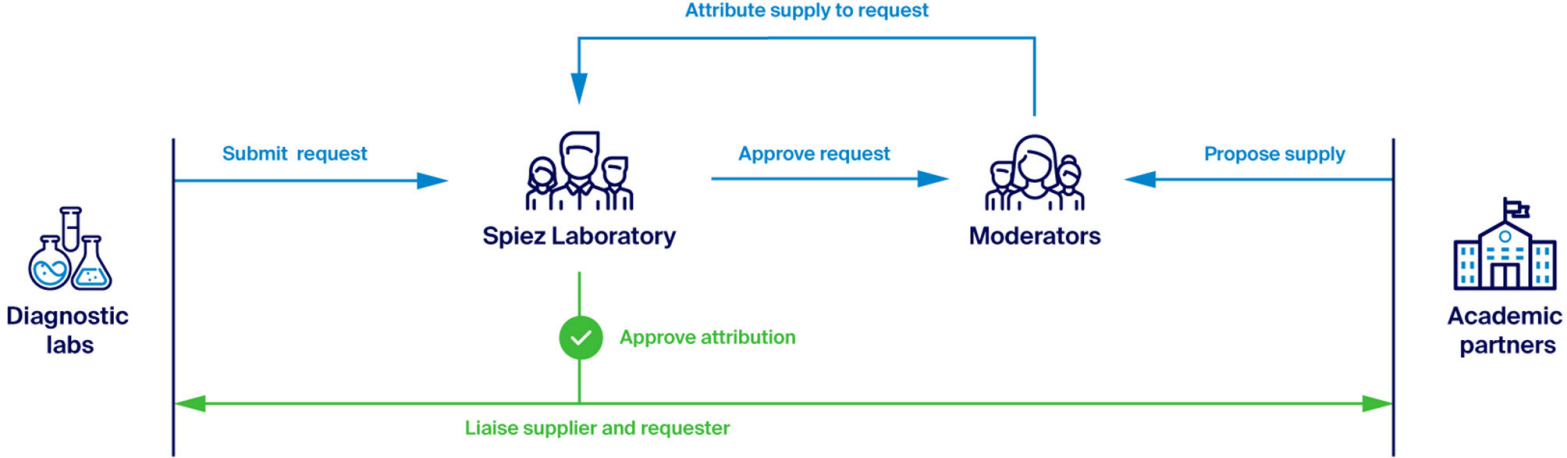
The Academic Resources for COVID-19 Platform (ARC): Actors and high-level workflow. ARC is designed to facilitate the sourcing of critical supplies needed by diagnostic laboratories from academic laboratories, while allowing a central decision-maker (Spiez Laboratory) to prioritize and approve individual agreements.

In one of its first proposed measures, the task force identified the possibility that academic research groups—particularly those in the biological and medical sciences—could share equipment, reagents, and consumables with hospitals and diagnostic laboratories. To make this process more effective, it was decided to set up a web-based platform that would allow academic research groups across Switzerland to respond to needs expressed by hospitals and diagnostic laboratories. The platform was eventually named the Academic Resources for COVID-19 (ARC) Platform. The ARC Platform is not the only initiative of this kind [see for example GetUsPPE, crowdfightcovid19, and the COVID-19 National Scientist Volunteer Database; (9, 10)]. However, the key distinguishing features of the ARC are the use of a controlled (but extensible) vocabulary allowing laboratories with supplies to gain a precise picture of current needs, and the implementation of a match-making logic under the supervision of a central decision-maker, with responsibility for ensuring that exchanges of resources are in line with the priorities dictated by the changing dynamics of the pandemic. Figure 1 illustrates the high-level workflow of ARC and the main actors.

The ARC Platform was developed in tight collaboration between groups of the two Swiss Federal Institutes of Technology [Blue Brain Project at the Ecole polytechnique fédérale de Lausanne (EPFL) and Eidgenössische Technische Hochschule—(ETHZ)], the Spiez Laboratory and Apptitude SA, a Swiss startup, as part of the task force's expert group “Exchange platform.” In the development the Agile methodology (https://agilemanifesto.org/) and continuous deployment were used to roll it out as quickly as possible, while maintaining the ability to quickly evolve the Platform when it was already in operation. The ARC Platform, based on the Django framework (https://djangoproject.com), is customizable and multi-user capable. In the hope that it may help other countries in their own fight against COVID-19 or future pandemics, we have made the ARC software available as open source software. Since roles and types of requests are highly configurable, use is not limited to the COVID-19 pandemic. We believe that the ARC Platform could also help in the response to other crises such as natural disasters.

## 2. Materials and Methods

### 2.1. Functional View

#### 2.1.1. Roles

ARC implements the concept of roles. This makes it possible to configure which user can perform which actions. Available functionality is presented to the user in the form of tailored dashboards and workflows. A user can have multiple roles.

The four principal roles are the following:

- **Requesters** submit a description of their needs, and indicate the urgency of their request from low to high priority. They can browse offers of supplies, but only see (anonymized) requests from other requesters if they also are suppliers. Requesters are typically admitted to the Platform on invitation. The Requester role is motivated by the functional requirements of diagnostic laboratories needing critical materials, such as equipment, consumables, reagents, or expertise.
- **Suppliers** can offer needed equipment, consumables, reagents, or expertise and can post details and quantities on the Platform. Suppliers can see the (anonymized) list of current requests and define their offers in terms of the taxonomy provided (see Section 2.1.2). This role is motivated by the functional requirements of academic groups reacting to the critical needs of diagnostic laboratories.
- **Validators** are able to approve the posting of requests and validate matches between requests and offers. Validators can see submitted requests, published requests, and all offers of supplies. Validators can override previously determined priorities and set requester identity to confidential. The Validator role is motivated by the functional requirements of Spiez Laboratory.
- **Moderators** are able to suggest matches between requests and offers of supplies in support of the Validator role. They can see all published requests and all offers of supplies. This role can be thought of as a delegation by the Validator to a group of domain experts. In practice Moderators come from a team of ETHZ postdoctoral researchers and PhD students supporting Spiez Laboratory. The Moderator role is motivated by the needs of this team.

ARC also implements an **Administrator** role. The Administrator manages users through a separate part of the Platform allowing adding/removing users and the assignment of roles.

#### 2.1.2. Taxonomy

One of the core concepts in the design of ARC is the use of a taxonomy for requests and offers of supplies. Online marketplaces typically provide basic categories such as “buy” and “sell” or categories of items. However, actual ads are typically not forced to use a controlled vocabulary. In the context of ARC, however, it is highly important to specify requests and offers precisely, for example to show that a consumable is really compatible with the equipment for which it is needed. Accordingly, ARC employs a customizable taxonomy determining the main classes of requests and guides requesters and suppliers to register their requests/offers in terms of a hierarchical identification scheme prescribed by the taxonomy. This taxonomy can be defined upfront. However, requester and suppliers can both propose new entries. It is then the role of the moderators to harmonize those proposed additions.

Given the goals of the Platform, the highest level in the taxonomy is the type of a request or offer. The Platform defines the following types:

- **Equipment**—large multi-use, permanent, physical items, or facilities needed for critical testing function (e.g., QPCR machines, diagnostic robots);
- **Consumables**—single or limited-used physical items found to be in short supply (e.g., nasal swabs, masks, test tubes);
- **Reagents**—typically, chemicals, solutions, or culture media that can be measured in continuous quantities (extraction and diagnostic kits are also included in this category);
- **Know-How**—knowledge needed to handle specific knowledge gaps or to identify suitable protocols for specific tasks (e.g., to purify or synthesize PCR primers used for testing);
- **Personnel**—trained personal (including volunteers) to assist in diagnostic testing

Each type additionally contains a number of subgroups (Categories). For example, categories for the Reagents type include: nucleic acid extraction reagents, PCR reagents, and culture media. Similarly, categories for the Consumables type include: personal protective equipment, swabs, test tubes, and pipette tips. See Table 1 for a complete overview of the first two levels of the taxonomy.

**Table 1:** Overview of the ontologies used to categorize requests and supplies.

**Types**	**Consumables**	**Reagents**	**Equipment**	**Personnel**
Categories	Medical instrument	Antibiotic	Diagnostic equipment	Technical advancements
	Specialized	Antifungal	Fabrication	Fabrication
	Plates	Media	Hood/biosafety cabinet	Logistics and policy
	Personal protective equipment	Molecular biology reagent	Nucleic acid extraction robot	Medical or emergency support
	Sealing film	Nucleic acid extraction	Medical equipment	Data analysis and modeling
	Waste containers	Primers/probes	PCR machine	
	Swab/sample collection	qPCR/PCR reagents	qPCR machine	
	Transport (shipping)	Sample collection kit	Specialized	
	Tubes			
	Reservoirs			

Below the level of category, users have to specify a Manufacturer. The manufacturer can be specified as “Generic”. While this was initially somewhat undesirable with regard to maintaining the taxonomy, it was necessary, as in its absence, similar but not exactly matching supplies would be submitted, and while well-meaning, these matches would often later be rejected, as they would break the Standard Operating Procedures (SOP) of the requesting diagnostics lab. This allowed requesting diagnostics laboratories to specify items for which the manufacturer may not matter—a typical example would be personal protective equipment, particularly gloves and masks, where any manufacturer is acceptable provided the glove material or mask rating is the same.

Below the manufacturer level, entries are identified by a specific name or reference number.

While the primary goal of the taxonomy was to speed up matching between offers of supplies and critical needs, the design also had a second goal, namely to identify situations where the substitution of one item for another would not break laboratory SOPs (in the case of latex gloves, for instance) or where one item could be substituted for another similar item in the event of a critical shortage.

#### 2.1.3. Requests

A *request* is the description of a need by a user with the Requester role (see Section 2.1.1) for equipment, reagents, consumables, know-how, or personnel or anything else that could be defined in the customizable taxonomy (see Section 2.1.2). To this end, the requester submits a request to the system by filling in the type, category, manufacturer, and name of the needed item following the taxonomy managed by the moderators. In addition, the requester indicates the quantity needed, the priority, and the sensitivity of the request.

The submission of the request will notify all Platform users with Validator role (see Section 2.1.1). Validators can monitor the incoming request through a dedicated dashboard where they can accept or reject it. The decision will notify the requester. If the request is accepted, moderators and other validators are also notified. Once validated, the request is available for the match-making workflow (see Section 2.1.5).

After a configurable expiration period, validators receive a notification, allowing them to decide if the request should be maintained or archived. This ensures that only relevant requests are maintained. If validators do not explicitly decide to maintain a request beyond the expiration period, it is automatically archived.

#### 2.1.4. Supplies

A *supply* can be equipment, reagents, consumables, know-how, personnel or anything else that could be defined in the customizable taxonomy (see Section 2.1.2). Supplies must be registered in the Platform by a user with the Supplier role. To this end, suppliers can login to the Platform, and view a list of requests, thereby gaining a sense of what is needed. Suppliers define supplies (requested or otherwise) through the Platform interface, following the taxonomy created by the moderators. The supplier also defines the pickup location for the supply.

To ensure end-to-end quality, the supplier is asked to enter only intact, complete and unopened supplies. To ensure the compatibility of complex equipment/consumables, the supplier is also asked to provide a catalog number, accurately identifying the resource. Quantities are specified in term of multiples of original boxes of the supply. If no term for the supply is listed in the taxonomy, the supplier can fill in a free form field, specifying the manufacturer and the name of the item. Moderators can then normalize the taxonomy accordingly. Normalization is performed on a regular basis.

Once an offer of a supply is submitted to the system, it is visible to the moderators and validators for match-making. To keep the database of supplies up to date, a periodic reminder e-mail is sent to suppliers to close the supply if it is no longer available for sharing.

#### 2.1.5. Matches

The central concept of ARC is the *match*—an explicitly created pairing of a Supply with a Request. The Platform automatically proposes matches based on the taxonomy-controlled compatibility of requests and supplies. Moderators can validate these automatic proposals or flag them as errors. Moderators typically monitor the Platform regularly or act on notification by the Platform.

**Figure 2:**
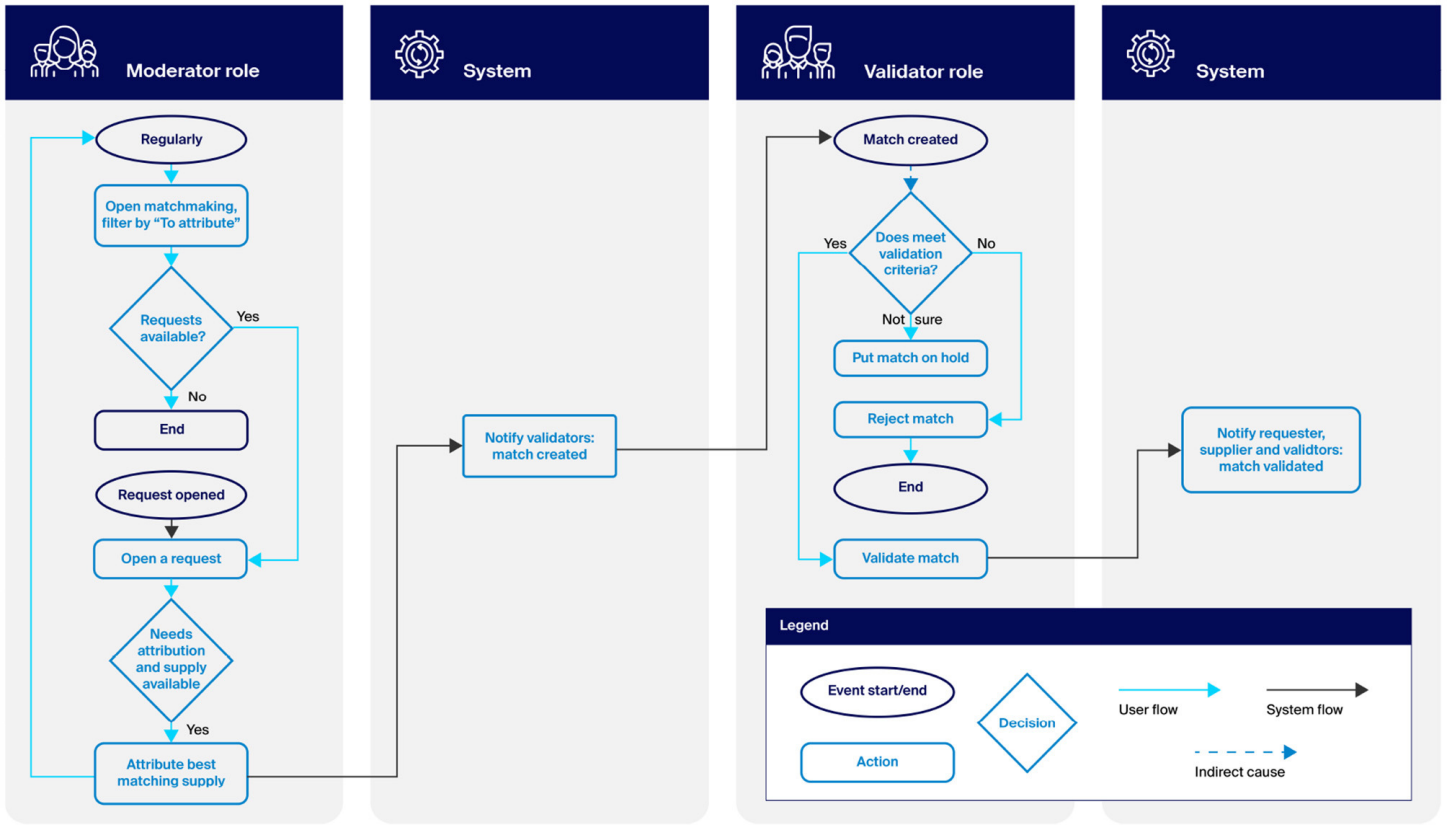
The match-making workflow.

Once a match is created, it will effectively put a lock on the supply so that it cannot be allocated to another request—much like reserving an airplane seat. Once created, matches are shown on the Validator's match-making dashboard. Validators can then accept or reject the match or keep it on hold. A single request may have multiple matches. This allows Validators to pick the most appropriate match (e.g., the match where the supply is geographically closest to the requester). If, for one reason or another, a preferred match does not go through, Validators can consider one of the others. The supplier and requester are notified only when the match is validated. In this case, they receive a pre-configured email with instructions. Figure 2 illustrates this workflow.

**Figure 3:**
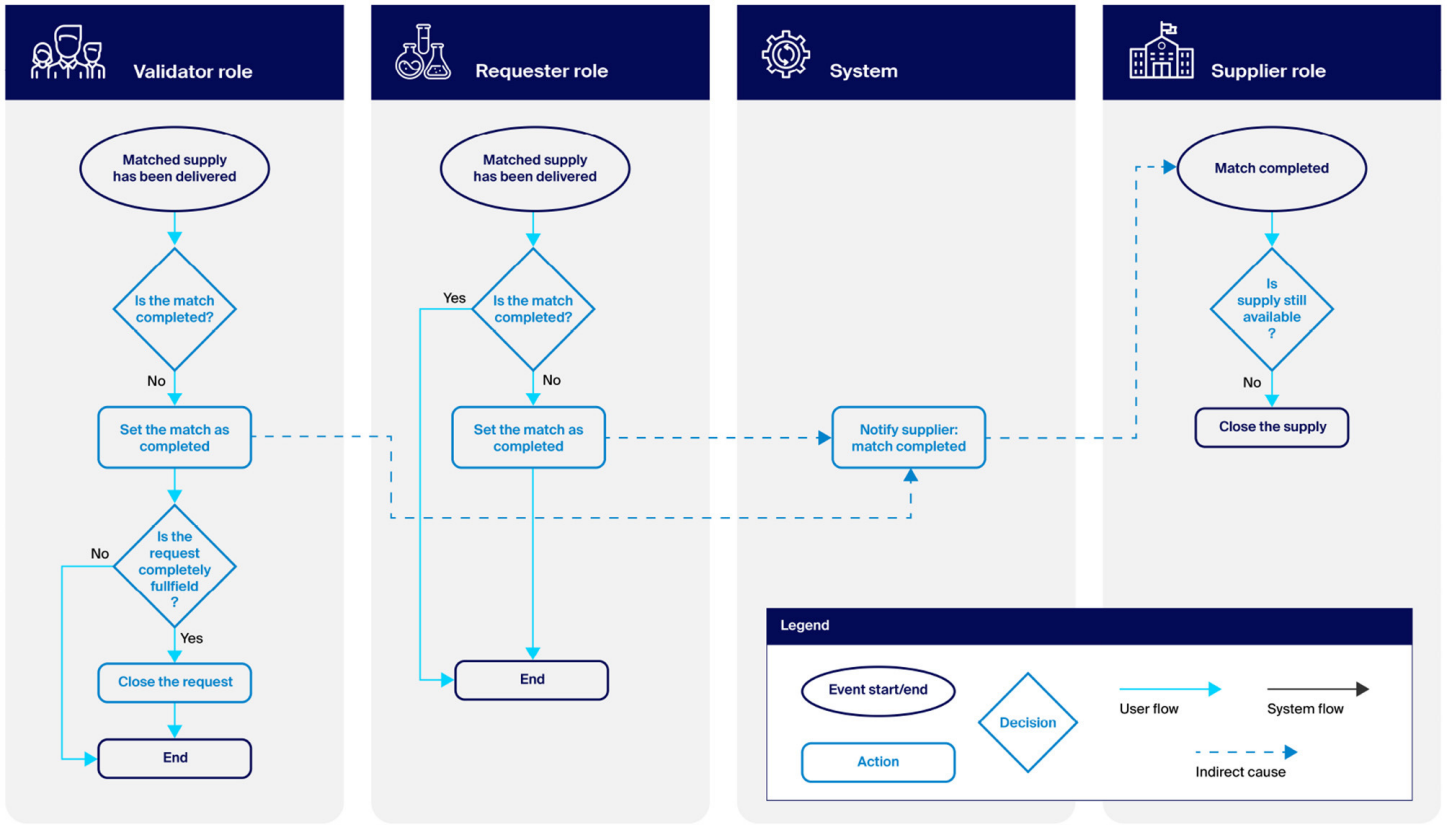
The match completion workflow.

Apart from the notification email, the Platform provides no specific functionality for shipping and handling. However, it does track when a match has been successfully completed (e.g., when a supply has been shipped to the requester). This makes it possible to maintain an up-to-date picture of which supplies are still available and which requests are still open. Confirmation that a match has been completed can come either from the requester or the validator. Once completion is confirmed, the supplier is notified, the supply is removed or reduced in quantity, and the request is closed (i.e., removed from the request dashboard). Figure 3 illustrates this workflow.

Lastly, the automated match-making capability relies on a well-defined taxonomy. While it is possible to register requests/supplies that are not in the taxonomy, the automated match-making ability will not be available in such a case. It is thus recommended that the definition of the taxonomy follows the evolution of the demand (see Section 2.4).

### 2.2. Implementation

#### 2.2.1. UX Design

The user experience (UX) with ARC is designed to be intuitive from the first connection, yet effective for use in everyday situations even under stressful circumstances. The Platform is structured with both high-level and detailed views. Lists of requests and supplies provide an overview of the general situation, while lists of matches facilitate the decision making and execution thereof. The detailed views allow users to focus on indicating resources and making matches.

To deliver ARC as quickly as possible, the design process consisted of rapid iteration loops each lasting a few days or a few hours. The design process involved tight collaboration with all project stakeholders, based on an evolving interactive prototype. To ensure relevance, and feasibility, end-users and technical experts both contributed to the early phases of the design.

#### 2.2.2. Platform Architecture

The ARC Platform is developed in Python with the Django framework, which enforces a standard code structure and offers useful features, including a powerful object-relational mapping (ORM) and automatic HTML form generation from database models. The development team used these features heavily. To keep the number and size of files manageable, the code is organized in four Django applications, each representing one of the main functionalities of the Platform.

- The *app* application contains the main page template with navigation and footer, general purpose pages (e.g., terms of service, landing page) and reusable HTML templates (e.g., styled form fields, modals). It also contains custom middleware to prevent access from machines other than the authentication proxy, and to show terms and conditions to users who have not yet given their approval.
- The *user* application contains the user model, user administration views, a single sign-on (SSO) login route, and an email/password login form.
- The *resources* application contains the models used for resource categorization including lists of types, categories, and manufacturers. As the editing of the ARC taxonomy is restricted to moderators, we used default Django admin views here.
- The *matchmaking* application contains views and forms for request/supply creation and validation.

**Figure 4:**
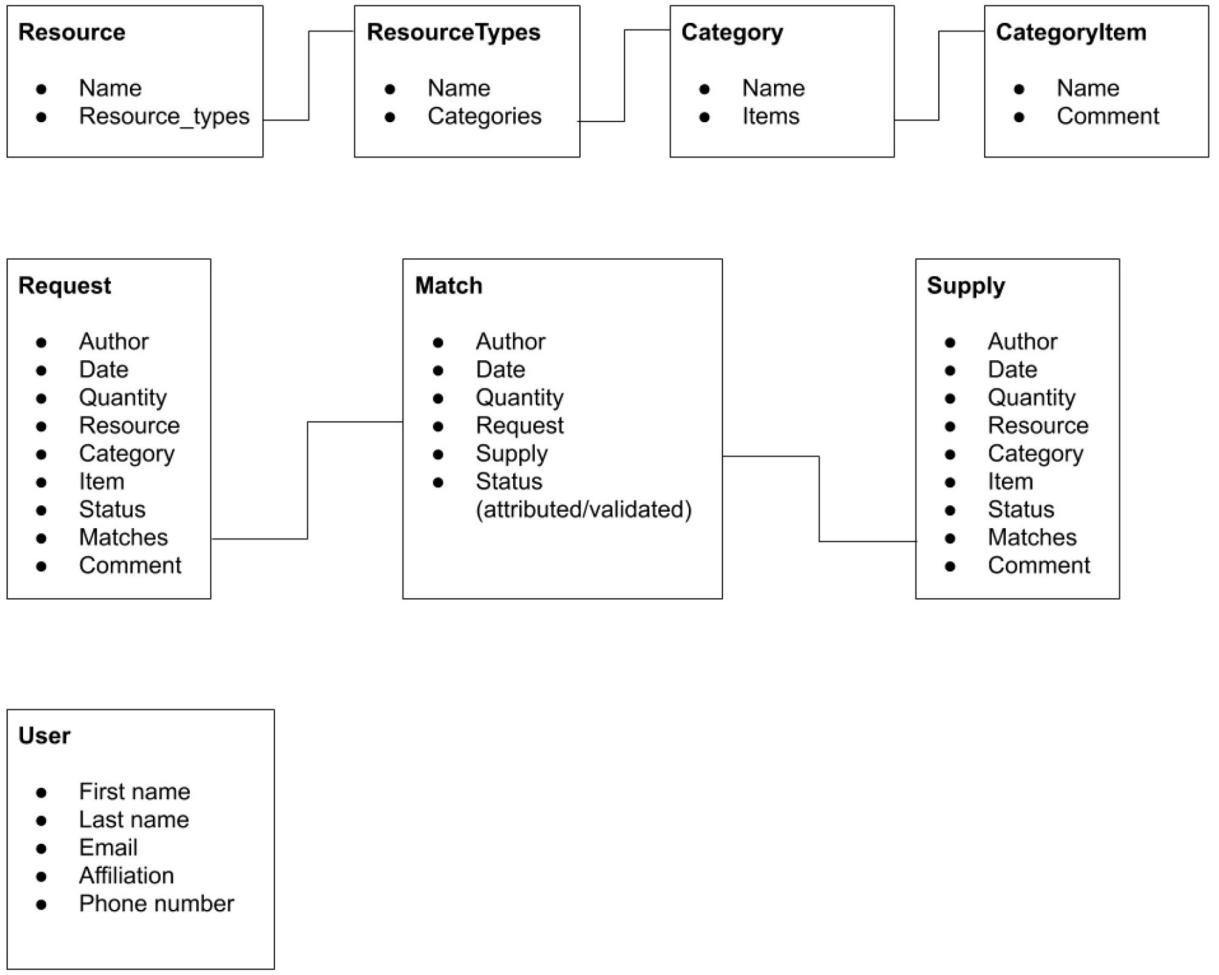
Data model for the ARC platform.

Figure 4 shows the data model for the application. The models are defined in the *models.py* files of their respective Django applications. In the user interface and this paper, the *Resource, ResourceTypes, Category*, and *CategoryItem* models are named *Type, Category, Manufacturer*, and *Item*.

One requirement of the system could not be satisfied with standard Django components. In the user interface, the request and supply forms need to show four selection dropdowns to select the type, category, manufacturer, and item. These fields have to be selected sequentially, using options which depend on the option selected for the previous field. A page reload between selections would have been unacceptable. We therefore had to implement this behavior dynamically, using JavaScript. To achieve this in a reusable manner, we used the Vue framework to create a dedicated component and rendered the dynamic data for the selection logic as JSON in the form pages.

#### 2.2.3. Authentication and User Management

Two complementary authentication mechanisms were implemented: (1) a SWITCHaai authorization and authentication infrastructure for suppliers, and (2) a Django-based authentication for other roles.

As the suppliers in our scenario are mainly Swiss academic partners, we benefited from the federated identity management services provided by SWITCHaai (https://www.switch.ch/aai/). SWITCHaai provides a simple unified login to web services based on the Shibboleth architecture. This avoids the need to register all possible academic users, as they are all managed by their home organizations. We registered our application to the SWITCHaai resource registry since the service provider integration is provided natively by Apache front-end server (https://cxf.apache.org/docs/saml-web-sso.html). The front-end server issues a SAML authentication request to the identity provider selected by the end user (the university the user chooses to authenticate in the login panel). It then processes the response, extracts the full name, affiliation and email of the end user, and passes the information to the Django application server. To replace SWITCHaai authentication with authentication by another custom SAML provider, all that needs to be done is to place the application behind a front-end server that intercepts the access to the application entry point and executes the custom authentication workflow to assert the identity of the user. The front-end server can then forward the necessary HTTP headers to the application server (see Section 3.2).

For users outside the SWITCHaai domain, we use Django-based authentication (using the Django middleware named AuthenticationMiddleware). Here, users are registered by the application and managed by an administrator. Users can be registered either in the Django shell or through the user tab in the user interface.

User roles can be set in the user tab of the Platform after a user logs on for the first time (in case of SWITCHaai) or on creation of the user account (in the case of Django-based authentication). By default, a user is given the role of a Supplier. Permissions are tested at different levels of the Platform based on the role of the user. This means that the user is not offered functionality irrelevant to his or her role and that only users with the appropriate role can perform certain actions.

### 2.3. Development and Deployment

#### 2.3.1. Staged Development Strategy for Rapid Deployment

In light of the growing crisis, an initial exchange platform needed to be put online as quickly as possible. While the development for the fully featured platform described in this work was initiated, a first platform (ARC1) was built based on Microsoft SharePoint. It became operational within 12 hours of the task force mandating the idea. This version implemented the requests and supplies as multi-user capable tables, however, left the matching as an offline activity to the moderators.

For the final platform (ARC2), the development started before the system was fully designed. A great level of flexibility to adapt to the evolution of the requirements over time was thus required from the team. To achieve this flexibility, the Agile development methodology was used with short daily meetings of the whole team (developers, designers, and project management) and multiple task-specific meetings between the concerned developers.

#### 2.3.2. Deployment

The final platform as described here was deployed as a Docker container (11). The container provides the Django-based application server and serves the client side code (HTML, javascript, and css files). The application is delivered through the Apache web server version 2.4 (https://httpd.apache.org/) using the shibboleth module version 3 for Apache. A full installation also requires a postgreSQL database to store platform data; the public-facing Platform (https://arc.epfl.ch) uses postgreSQL 9.3.5 with OpenShift Origin 3.11 as the container application platform. Two additional (internal) environments provide a test environment where validators and moderators can assess the latest features, and a qualification environment for testing.

### 2.4. Customization

As can be seen in Table 2, customizing an instance of the ARC Platform for a different set of requests and supplies can be done directly from within the running platform when new types of requests arise. This makes it possible for others to deploy a similar platform in their own countries, with only minor adjustments. For example, such a platform could be used to support hospitals exchanging equipment or to coordinate crisis responses among actors in local, regional and central governments. The key feature which all these use cases have in common is the presence of a specific actor responsible for prioritizing requests, making decisions and maintaining an orderly crisis response. This distinguishes the ARC technology from typical platforms for classified ads, where the only actors are the requesters and the suppliers. Use cases which require this kind of centralized supervision can be readily addressed through customization.

**Table 2:** Overview of readily customizable features of the platform.

**Taxonomy customization**	
Add a new resource	With superadmin role, click on Resources; in the Home>Resources breadcrumb, click on add resource.
Add a new manufacturer	With superadmin role, click on Resources; in the Home>Resources breadcrumb, click on add manufacturer.
Add a new category	With superadmin role, click on Resources; in the Home>Resources breadcrumb, click on add category.
Add a new category item	With superadmin role, click on Resources; in the Home>Resources breadcrumb, click on add category item.
**Mailing template customization**	
For the following template:	In the code repository, under arcv2_platform/templates/email, edit and redeploy the corresponding template.
**Automation customization**	
Request expiration delay (in days)	In the arcv2_platform/config/common.py of your deployment, modify the APP_REQUEST_EXPIRATION_DAYS property.
**Authentication system customization**	
Use a custom authentication	Place the application server behind a front end server that intercepts the access to the application entry point and executes the custom authentication workflow to assert the identity of the user. Forward the following HTTP headers to the application server: “Remote-User,” “Givenname,” “Surname,” “Homeorganization,” “Telephonenumber.”
**Look and feel customization**	
Changing web page layout	In the code repository, edit arcv2_platform/templates/layout.html and redeploy.
Changing web page style	In the code repository, edit the arcv2_platform/static/src/scss/styles.scss file and redeploy.

Of course, more involved use cases may require more substantial extensions to the source code. For example, an application may require a geographic localization function to match open requests to close-by supplies or integration with messaging systems such as text messages on mobile phones. Since the application code is available as open source software, such extensions are always possible, though they may require some software engineering.

## 3. Results

**Figure 5:**
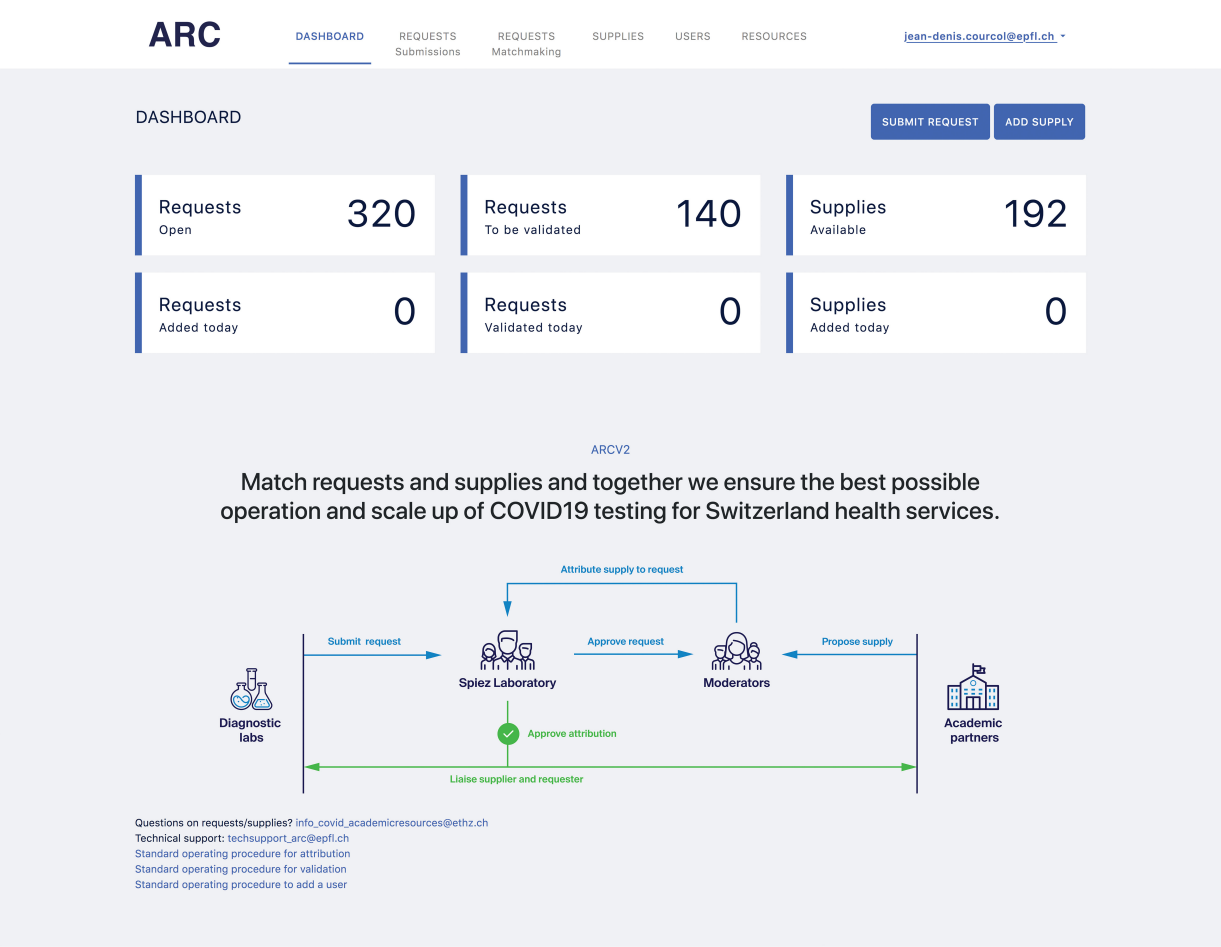
Screenshot of the Academic Resources for COVID-19 Platform operated in Switzerland under the URL: https://arc.epfl.ch/.

The Academic Resources for COVID-19 Platform as it is currently deployed in Switzerland (https://arc.epfl.ch) is shown in Figure 5. The screen shot shows the main dashboard of ARC as seen by users. The dashboard provides a high-level overview of the current status of requests and offers of supplies.

In Switzerland, Spiez Laboratory, the Swiss Federal Institute for NBC Protection, was mandated by the Federal Council to establish and maintain situational awareness with respect to all COVID-19 testing laboratories in Switzerland. In the terminology of this work, Spiez Laboratory is the Validator. At the height of the crisis, Spiez Laboratory was supported by a group of Moderators from ETHZ, one of the two Swiss Federal Institutes of Technology.

Requesters are Swiss medical diagnostic laboratories comprising University Hospital Laboratories (UHL), Hospital Laboratories (HL), and Private Laboratories (PL). From the time the pandemic began, at the end of February 2020, until June 2020, when most lockdown measures were lifted, the number of laboratories with SARS-CoV-2 diagnostic capabilities rose gradually to almost 80.

Suppliers were academic laboratories across Switzerland in a position to contribute resources, for example, groups working in biological, chemical, or pharmaceutical research. To identify potential suppliers, the moderators inspected the websites of relevant departments and identified relevant groups (18 departments/institutes from across the majority of the Swiss universities). Other academic laboratories (several hundreds) were alerted to the existence of ARC by an email which provided them with information about its goals, and instructions on how to contribute.

At launch (March 20, 2020), the initial version of the Academic Resources for COVID-19 Platform was populated with more than 300 requests for items ranging from basic consumables to PCR equipment. These requests were established by Spiez Laboratory through ongoing monitoring and direct querying of the diagnostics laboratories. These initial requests were used as the basis for the initial taxonomy (see Section 2.1.2). In the first three weeks since its launch, ARC handled nearly 400 offers from academic laboratories, 75% of which were submitted within the first week of operation. Offers came from more than 150 unique users from across Switzerland. It was possible to match about 55% of the offers with the requests, through manual curation by a team of moderators. The remaining 45% were categorized as unsolicited offers. Most of the unsolicited offers (43%) were offers of personnel. Spiez Laboratory validated matches and supervised the logistics of making the resources available to hospitals and diagnostic laboratories.

On April 24th (five weeks after the launch of the initial version of the platform), all requests, offers, and matches were transferred seamlessly to the new, custom-built ARC Platform, where they were handled from then on.

After a period of calm (mostly concurrent with a respite in the evolution of the pandemic in Switzerland), the ARC Platform was put on alert again in October 2020. At this point, the streamlined workflows and new notification system allowed operating the platform with a small team of few (less than five) persons working part-time and a reserve of moderators and validators that could be activated in case of need.

## 4. Discussion

In times of national crisis, resources for relief efforts are often in short supply. Frequently, countries receive international help from neighboring countries and others further afield, with recent examples being the Nepal Earthquake (https://www.bbc.com/news/world-asia-32477180) or the Australian fires (https://www.smh.com.au/politics/federal/call-for-help-international-response-to-australian-fires-20200106-p53p5r.html). In stark contrast, when the crisis is global and supply chains are strained worldwide, external help may not be available. At this point, it becomes necessary to find new and innovative solutions, including ones limited to one's own country. This is what the ARC Platform was designed to contribute to. Although the pool of resources available within a country is limited, the optimal allocation of these resources to the management of the crisis can play an important role in bridging gaps in the supply chain at moments of acute need, buying time for the development of other solutions: working together with international suppliers, expanding production capabilities, or repurposing the production chains of suppliers in neighboring sectors.

As a crisis develops, entropy increases, not only due to the crisis itself but also to the huge number of well-intentioned initiatives the crisis sets in motion. At this point, it becomes critically important to reduce the entropy by channeling everyone's efforts and initiatives into a single, comprehensive, well-coordinated, fact-based relief effort. The ARC Platform provides a space where needs and offers can be brought together, coordinated, prioritized, and validated in line with the best available knowledge about the current development of the crisis at a national and local level.

In the beginning of the first wave of the COVID-19 pandemic in Switzerland, where diagnostic testing had to be ramped up in a very short time, even basic molecular biology laboratory equipment that could previously simply be ordered in a catalog was suddenly not available and threatened to put a stop to testing workflows at individual sites or prevent the ramp up. In this situation, the resources available in academic laboratories—despite also being limited—were a temporary relief. During the first wave, the ARC Platform handled close to 300 requests and 400 offers from about 150 unique users and could match about 55% of the offers with requests.

After the first wave had flattened, supplies could mostly be restocked through regular supply channels. Nonetheless, the ARC Platform was put again on alert in October 2020, shortly after the beginning of the second wave in Switzerland. While this phase is not over yet, already two things have become obvious. On the one hand, the resources required by diagnostic laboratories have changed in nature due to the availability of novel test kits and are also more numerous due to the continuous high-throughput operation. These resources are less likely to be sourced in quantity from the academic sector. On the other hand, the investment into developing a more tailor-made version of the ARC Platform proved valuable as the monitoring and operation of the Platform is more streamlined and requires far fewer people to attend to it now. The ARC Platform is thus not a regular supply chain but it is a low-overhead, bridging measure to remedy shortages of specialized components, resources, and skills typically found in the academic sector. One salient ongoing example is the current worldwide shortage in pipette tips, a simple laboratory supply that is often stocked in large quantities in academic laboratories and which is currently being successfully made available to diagnostic laboratories trough the ARC Platform.

From a development point of view, the strategy to build two platform instances, using the gained experience with the first to inform the design and development of the second one proved beneficial. The ultra-rapid development, deployment, and operation of the initial Platform (less than 1 day) provided an immediate solution. The more involved, from-scratch development, quality assurance, deployment and production of the final ARC Platform (within several weeks—still rapid compared to a typical IT development project) provided a more effective, user friendly and comprehensive long-term solution.

Importantly, the development of the ARC platform was only possible due to tight collaboration between professionals in government, academia, a private start-up and willingness in the Swiss academic community to contribute resources. In this setting, it was essential to create links and establish trust between the actors, who in many cases had not collaborated previously. This task was greatly facilitated by the clear mandates the Swiss government had given to Spiez Laboratory and to the Swiss National COVID-19 Science Task Force. The same clear mandate also made it easier for individuals (e.g., the post-docs and PhD who volunteered as moderators) and organizations who committed resources for COVID-19 relief efforts (e.g., ETHZ and EPFL's Blue Brain Project) to engage in work far removed from their normal missions. Ultimately, the value of a platform like ARC depends on the willingness of academic groups to provide their resources for a crisis response.

Looking beyond, we believe that the ARC Platform concept can provide relief in other countries affected by COVID-19 for the purpose of sourcing specialized resources such as essential material goods, infrastructure (e.g., also space) and personnel from the academic sector, keeping in mind that this is a bridging remedy for sudden shortages and that, for sustained high-throughput testing, intact supply chains are ultimately required. Deploying a similar platform in another country is straight forward as the necessary software is freely available as open source. The operation of such a platform benefits from the streamlining of the workflows as described in this study and can be ensured by a small team of few (less than 5) persons working part-time. Only when large numbers of requests (hundreds and more) need to be matched or if the types of requests are changing rapidly (requiring particular attention to curate them into the taxonomy) may a larger team be required.

Ultimately, the platform is not limited to the types of actors as implemented in its current usage in Switzerland or to the current crisis. For example, diagnostic laboratories could not only be *requesters* but also *suppliers*, effectively turning the ARC technology into an exchange and distribution solution. Similarly, the supplier role could be opened to commercial providers turning the technology into a specialized market place for COVID-19 testing supplies. Lastly, due to its modifiable taxonomy the technology can also be applied to other contexts such as the exchange of hospital equipment or of humanitarian relief supplies. The ARC Platform technology can bring value to any similar application, where the entropy of the crisis is nefarious to the proper prioritization and successful matching of requests and supplies of rare essential goods, making the implementation of an oversight indispensable.

## Data Availability Statement

The web application of the ARC Platform is available as open source under a GPLv3 license; it can be accessed on GitHub under the URL https://github.com/bluebrain/arc-platform.

## Author Contributions

FS, RS, and CM conceptualized and led the study. J-DC, CI, ZL, and MM co-led aspects of the study. J-DC led the solution development. CI led the solution validation. ZL led the workflow integration. MM led the software development of ARC2. RS and SB conceptualized and led the work for ARC1. FS, RS, SB, HM, and AP contributed to the scoping of the study and funding acquisition. ZL, CM-P, FP, MP, UP, JS, and JW performed lead moderator duties, curating the taxonomy, managing matches, interfacing with suppliers and validators, organizing and supervising the overall moderator group. DB, EC, MD, BR, and MV performed moderator duties. J-DC, MM, PG, and MG performed the software development and validation of the ARC2 platform. BC performed the UX design of the ARC2 platform. KK performed the software development of the ARC1 platform. FS, RS, CM, and J-DC wrote the manuscript. MM, BC, CI, ZL, EC, MG, CM-P, FP, MP, JS, MV, and JW contributed to the manuscript. All authors gave feedback to the manuscript.

## Conflict of Interest

The authors declare that the research was conducted in the absence of any commercial or financial relationships that could be construed as a potential conflict of interest.
